# Genomic Epidemiology of Multidrug-Resistant *Escherichia coli* and *Klebsiella pneumoniae* in Kenya, Uganda, and Jordan

**DOI:** 10.3201/eid3014.240370

**Published:** 2024-11

**Authors:** Denis K. Byarugaba, Tamer S. Osman, Omar M. Sayyouh, Godfrey Wokorach, Collins K. Kigen, James W. Muturi, Vanessa N. Onyonyi, Mayar M. Said, Salwa A. Nasrat, Mahmoud Gazo, Bernard Erima, Stephen Alafi, Hope O. Kabatesi, Fred Wabwire-Mangen, Hannah Kibuuka, Anjali P. Sapre, Katelyn V. Bartlett, Francois Lebreton, Melissa J. Martin, Evelyn W. Mahugu, Hunter J. Smith, Lillian A. Musila

**Affiliations:** Makerere University College of Veterinary Medicine, Kampala, Uganda (D.K. Byarugaba); Makerere University Walter Reed Project, Kampala (D.K. Byarugaba, G. Wokorach, B. Erima, S. Alafi, H.O Kabatesi, F. Wabwire-Mangen, H. Kibuuka); US Naval Medical Research Unit EURAFCENT, Cairo, Egypt (T.S. Osman, O.M. Sayyouh, M.M. Said, S.A. Nasrat); Muni University, Arua, Uganda (G. Wokorach); Kenya Medical Research Institute/Walter Reed Army Institute of Research Africa, Nairobi, Kenya (C.K. Kigen, J.W. Muturi, V.N. Onyonyi, L. Musila); Kenya Medical Research Institute, Kisumu City, Kenya (C.K. Kigen, J.W. Muturi, V.N. Onyonyi, L.A. Musila); Ministry of Health, Amman, Jordan (M. Gazo); Makerere University School of Public Health, Kampala (F. Wabwire-Mangen); Walter Reed National Military Medical Center Multidrug-Resistant Organism Repository and Surveillance Network, Silver Spring, Maryland, USA (A.P. Sapre, K.V. Bartlett, F. Lebreton, M.J. Martin); General Dynamics Information Technology, Silver Spring (E.W. Mahugu); US Military Armed Forces Health Surveillance Division, Silver Spring (E.W. Mahugu, H.J. Smith)

**Keywords:** Antimicrobial resistance, *Escherichia coli*, *Klebsiella pneumoniae*, multidrug resistance, high-risk clones, bacteria, Kenya, Uganda, Jordan

## Abstract

Surveillance of antimicrobial resistance in Kenya, Uganda, and Jordan identified multidrug-resistant high-risk bacterial clones: *Escherichia coli* sequence types 131, 1193, 69, 167, 10, 648, 410, 405 and *Klebsiella pneumoniae* sequence types 14, 147, 307, 258. Clones emerging in those countries exhibited high resistance mechanism diversity, highlighting a serious threat for multidrug resistance.

Global transmission of high-risk pandemic clones of gram-negative bacteria presents a serious threat to human health and complicates bacterial disease management, resulting in high illness and death rates and an enormous economic burden on healthcare systems (*1*). The pathogens are characterized by resistance to multiple classes of antimicrobial drugs, carriage of virulence genes, transmissibility to humans and animals, and global distribution. The negative effects of antimicrobial-resistant infections in terms of gross domestic product and disease burden will be disproportionally borne by low- and middle-income countries (*2*,*3*).

Global high-risk clones are of particular concern because they are multidrug resistant, can persist in hosts, are highly pathogenic, can have a fitness advantage, and can transfer easily between hosts (*4*,*5*). Using an antimicrobial resistance (AMR) surveillance program spanning 10 years (2012–2022), we describe the population structure and features of high-risk multidrug-resistant (MDR) *Escherichia coli* and *Klebsiella pneumoniae* in Kenya, Uganda, and Jordan. 

## Methods

We examined the population structure of MDR isolates (defined as resistance to >3 classes of antimicrobial drugs) (*6*) from Kenya, Uganda, and Jordan (Appendix Figure 1) during 2012–2022, collected through the US Armed Forces Health Surveillance Division, Global Emerging Infections Surveillance program. Our study followed an active surveillance approach (with additional passive isolates in Kenya only), and according to the Centers for Disease Control and Prevention definition, infections were either healthcare-associated or community-acquired (Table) (*7*). 

**Table:** Demographic and clinical characteristics of patients from whom isolates were collected in study of genomic epidemiology of multidrug-resistant *Escherichia coli* and *Klebsiella pneumoniae* in Kenya, Uganda, and Jordan

Variable	*Escherichia coli,* no. (%)				*Klebsiella pneumoniae*, no. (%)		
	Kenya, n = 430	Uganda, n = 207	Jordan, n = 148		Kenya, n = 97	Uganda, n = 69	Jordan, n = 212
Age groups, y							
0–4	7.2 (31)	1.9 (4)	19.6 (29)		7.2 (7)	7.2 (5)	26.9 (57)
5–9	1.9 (8)	0	5.4 (8)		0	1.4 (1)	4.7 (10)
10–17	2.1 (9)	3.4 (7)	4.1 (6)		3.1 (3)	0	4.2 (9)
18–49	61.4 (264)	67.6 (140)	22.3 (33)		60.8 (59)	58 (40)	22.6 (48)
>50	27.4 (118)	24.2 (50)	45.9 (68)		28.9 (28)	27.5 (19)	41.5 (88)
Not available	0	2.9 (6)	2.7 (4)		0	5.8 (4)	0
Sex							
M	47.4 (204)	37.7 (78)	59.5 (88)		60.8 (59)	56.5 (39)	75.0 (159)
F	51.9 (223)	62.3 (129)	40.5 (60)		39.2 (38)	42.0 (29)	25.0 (53)
Not available	0.7 (3)		0		0	1.4 (1)	0
Infection type							
CAI	81.4 (350)	48.8 (101)	48.0 (71)		68.0 (66)	33.33 (23)	14.2 (30)
HAI	15.8 (68)	42.5 (88)	52.0 (77)		28.9 (28)	56.52 (39)	85.8 (182)
Not available	2.8 (12)	8.7 (18)	0		3.1 (3)	10.14 (7)	0
Year of isolation							
2011	0	0	0		0	0	0.5 (1)
2012	0	0	6.8 (10)		0	0	9.0 (19)
2013	0	1.0 (2)	16.2 (24)		0	0	14.2 (30)
2014	0	0	18.2 (27)		0	0	7.1 (15)
2015	7.7 (33)	10.1 (21)	26.4 (39)		4.1 (4)	7.2 (5)	34.0 (72)
2016	4.2 (18)	9.7 (20)	21.6 (32)		3.1 (3)	21.7 (15)	21.2 (45)
2017	7.4 (32)	7.7 (16)	3.4 (5)		10.3 (10)	10.1 (7)	10.8 (23)
2018	25.6 (110)	5.8 (12)	4.1 (6)		29.9 (29)	5.8 (4)	0.9 (2)
2019	19.8 (85)	4.3 (9)	3.4 (5)		17.5 (17)	5.8 (4)	2.4 (5)
2020	6.7 (29)	13.5 (28)	0		5.2 (5)	7.2 (5)	0
2021	15.3 (66)	22.2 (46)	0		21.6 (21)	30.4 (21)	0
2022	13.3 (57)	25.6 (53)	0		8.2 (8)	11.6 (8)	0
Sample type							
Wound/skin	49.1 (211)	10.6 (22)	11.5 (17)		71.1 (69)	2.9 (2)	0.9 (2)
Urine	39.3 (169)	57.5 (119)	34.5 (51)		20.6 (20)	39.1 (27)	11.8 (25)
Blood	0.2 (1)	1.0 (2)	20.3 (30)		0.0	2.9 (2)	20.8 (44)
Pus	8.4 (36)	29.5 (61)	0		7.2 (7)	43.5 (30)	0
Throat	0.5 (2)	0	0		0	0	0
Respiratory	0.0 (2)	0	33.8 (50)		0	2.9 (2)	66.5 (141)
Other	2.1 (9)	1.4 (3)	0		1.0 (1)	8.7 (6)	0
Not available	0.5 (2)	10.6 (22)	0		0	0	0

* CAI, community-acquired infection; HAI, healthcare-associated infection.

During 2012–2019, in Jordan, the Naval Medical Research Unit EURAFCENT, together with the Jordan Ministry of Health, collected 148 *E. coli* and 212 *K. pneumoniae* isolates from 9 hospitals (Appendix). During 2012−2022, in Kenya, the Walter Reed Army Institute of Research-Africa and the Kenya Ministries of Health and Defense collected 430 *E.coli* and 97 *K. pneumoniae* isolates from 12 hospitals. Also during 2012−2022, in Uganda, Makerere University Walter Reed Project, together with the Uganda Ministry of Health and Ministry of Defense, collected 207 *E.coli* and 69 *K. pneumoniae* isolates from 4 hospitals. Together, those collections resulted in a total of 785 *E. coli* and 378 *K. pneumoniae* MDR clinical isolates analyzed in our study (Appendix). The isolates were collected from patients 0.1–104 years of age and from different sources, including wounds (n = 323), urine (n = 411), blood (n = 79), pus (n = 134), respiratory tract (n = 195), and others (Table). To identify MDR strains for further characterization through whole-genome sequencing, we tested susceptibility to a panel of different classes of antimicrobials by using disk diffusion and the VITEK2 system (bioMérieux, https://www.biomerieux.com) in accordance with Clinical and Laboratory Standards Institute guidelines (*8*).

We subjected all MDR *E.coli* and *K. pneumoniae* isolates to whole-genome sequencing and de novo assemblies as previously described (*9*) and deposited the data in GenBank (BioProject accession nos. PRJNA955428, PRJNA1015582, PRJNA1076681, PRJNA1076682, PRJNA1078230, PRJNA1078534, PRJNA1078535). We assessed the population structure by using core-genome multilocus sequence typing and species-specific minimum spanning trees as previously described (*9*). 

## Results

**Figure 1:**
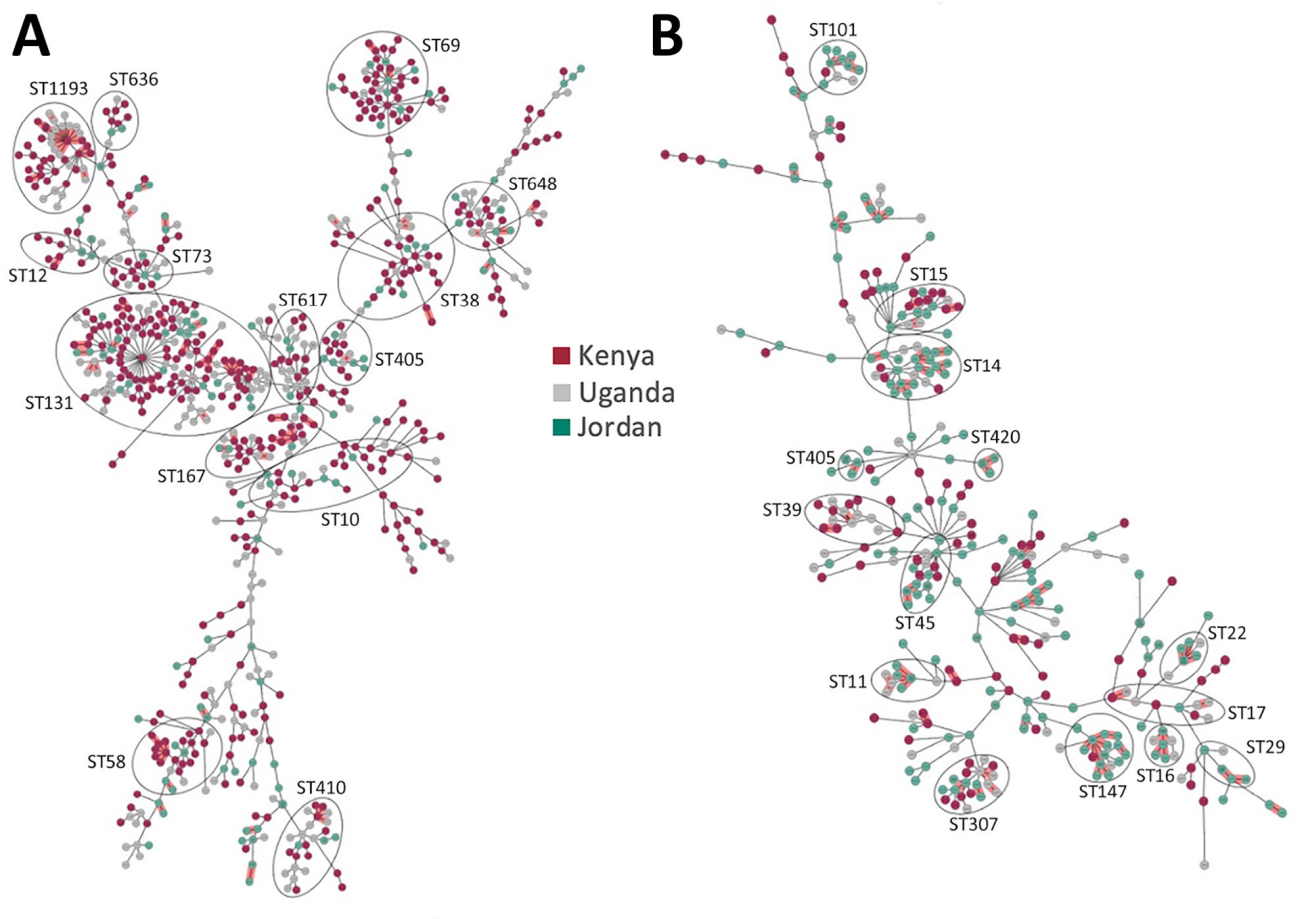
Population structure and diversity of high-risk *Escherichia coli* and *Klebsiella pneumoniae* sequence types across Kenya, Uganda, and Jordan. Minimum-spanning trees of *E. coli* (n = 785) and *K. pneumoniae* (n = 378) isolates are based on core-genome multilocus sequence typing. Each node represents an isolate; dominant STs are indicated in circled clusters. Branch length between nodes is proportional to the allelic differences between nodes. Purple indicates isolates from Kenya, gray from Uganda, and green from Jordan. ST, sequence type.

The 785 *E. coli* genomes represented 124 sequence types (STs), of which 20 (16.1%) were shared between countries (Figure 1). For *E. coli*, the dominant ST was ST131 (Figure 1) in all 3 countries (Kenya 21.6%, n = 93; Uganda 21.3%, n = 44; and Jordan 16.9%, n = 25), collectively representing 20.6% (n = 162). The global high-risk clones (STs 131, 1193, 167, 69, 38, 10, 648, 410, 405, 73, 12, 117, 127, 95, and 393) constituted 62.4% (490/785) of all isolates. Evolution of the high-risk strains over the years was noted; in 2020, ST1193 became dominant in Kenya and Uganda, and no isolates were available from Jordan after 2020 (Appendix Figure 2). ST131 isolates decreased dramatically in Kenya in 2020 and in Uganda in 2018 and 2019; ST10 peaked in Jordan in 2012, in Kenya in 2018–2020, and in Uganda in 2020, after which it declined. ST648 sporadically appeared annually across all countries. The dominant *E. coli* phylogroups in all countries were B2, A, D, and B1, which comprised 90% of the isolates; B2 was the most dominant at 39.5%.

Similarly, genetic diversity of *K. pneumoniae* was high. There were 123 distinct STs, and only 11 (8.9%) STs were shared across the 3 countries (Figure 1). Jordan and Uganda had 75 distinct STs each, and Kenya had 37 STs. No clear evolutionary patterns of STs were observed over the years; STs appeared sporadically in different years except for ST420, which emerged in Uganda from 2020 to become a dominant ST, and ST14, which was the dominant strain in Jordan during 2013–2015. The high-risk clonal groups (CGs; 14, 15, 16, 101, 147, 307, 23, 65, 231, 258, 86) were detected and represented in 29.1% of the isolates. The high-risk CG14 (ST14) and CG147 (ST147) were very dominant in Jordan; CG15 and CG55 were exclusive to Kenya, and the global high-risk clone CG258 (ST258) was only in Jordan.

**Figure 2:**
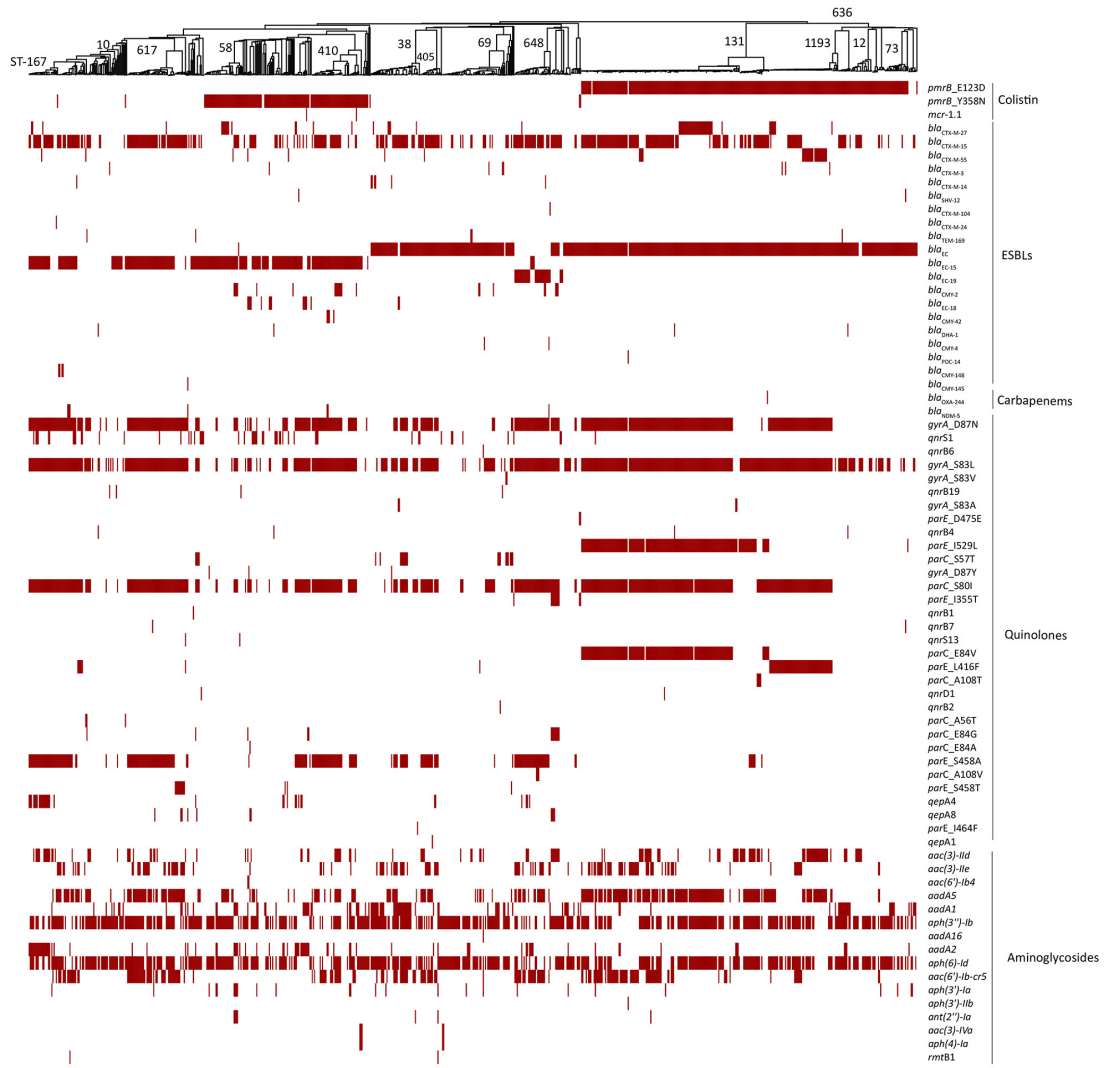
Comprehensive distribution of antimicrobial-resistance genes in 785 *Escherichia coli* isolates from Kenya, Uganda, and Jordan. Antimicrobial-resistance genes associated with nonsusceptibility to various antibiotic classes (polymyxins, third- and fourth-generation cephalosporins, carbapenems, phenicols and quinolones, and aminoglycosides) for each isolate are labeled for presence (red) or absence (white). The presence or absence of gene(s) is mapped onto a neighbor-joining tree curated from its minimum-spanning tree. The major high-risk STs are labeled on the neighbor-joining tree. ST, sequence type.

**Figure 3:**
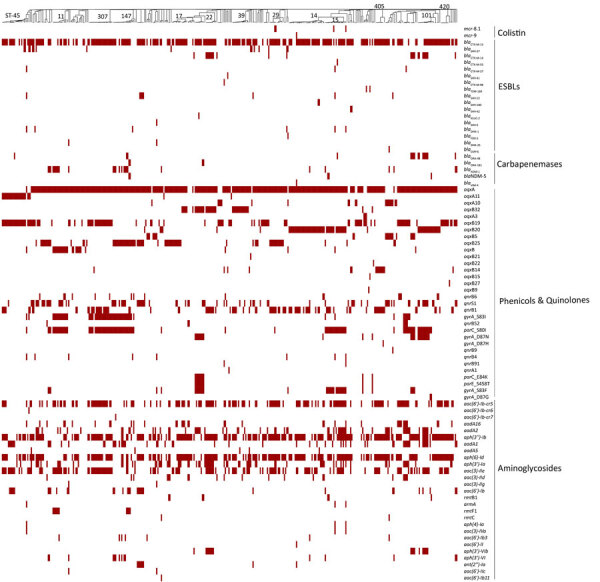
Comprehensive distribution of antimicrobial-resistance genes in 378 *Klebsiella pneumoniae* isolates from Kenya, Uganda, and Jordan. Antimicrobial-resistance genes associated with nonsusceptibility to various antibiotic classes (polymyxins, third- and fourth-generation cephalosporins, carbapenems, phenicols and quinolones, and aminoglycosides) for each isolate are labeled for presence (red) or absence (white). The presence or absence of gene(s) is mapped onto a neighbor-joining tree curated from its minimum-spanning tree. The most prevalent STs are labeled on the neighbor-joining tree. ESBLs, extended-spectrum β-lactamases; ST, sequence type.

We analyzed whole-genome sequences for resistance determinants by using AMRFinderPlus (*10*) and ARIBA (*11*) and iTOL software version 6.8.1 (https://itol.embl.de) for visualization (*12*), as previously described (*9*). *E. coli* had 145 (Figure 2) and *K. pneumoniae* had 200 (Figure 3) diverse resistance determinants for various classes of antimicrobial drugs. Among the resistance determinants of concern were the acquired extended-spectrum β-lactamases (ESBLs), mainly because of carriage of the *bla*_CTX-M-15_ gene, identified in 50.8% of *E. coli* isolates and 68.8% of *K. pneumoniae* isolates, distributed in different STs (Figure 3). For *E. coli,* most (28.5%) ESBLs were in ST131, and the *bla*_CTX-M-27_ allele was detected in 15% of the isolates. Carbapenem resistance was detected more in *K. pneumoniae* than in *E. coli*. In *K. pneumoniae*, carbapenemase genes were detected in 47 (12%) isolates, 43 of which were from Jordan; some isolates were co-harboring multiple carbapenemases, other resistance determinants, or both, including ESBL genes. Carbapenem resistance genes in Jordan included *bla*_NDM-1_ (11.3%), *bla*_OXA-48_ (7.3%), *bla*_OXA-181_ (0.9%), *bla*_NDM-5_ (0.9%), and *bla*_VIM-4_ (0.3%) (Figure 3). Isolates from Uganda carried *bla*_OXA-181_ (2.9%) and *bla*_NDM-5_ (1.4%). *bla*_NDM-1_ and *bla*_NDM-5_ were detected in isolates from Kenya, both at 2.1%. Four isolates, all from Jordan, belonged to lineage ST147 and were of serotype K64:O2a that co-carried *bla*_NDM-1_ and *bla*_OXA-48_ (n = 2) or *bla*_NDM-5_ and *bla*_OXA-181_ genes (n = 2); 1 isolate from ST23, of serotype K1:01, also carried *bla*_NDM-1_ and *bla*_OXA-48_. In *E.coli*, carbapenemase genes were detected in 8 isolates: *bla*_NDM-5_ (n = 7) and *bla*_OXA-244_ (n = 1) (Figure 2). Four isolates carrying *bla*_NDM-5_ co-carried *bla*_CTX-M-15_, belonging to lineages ST167 (n = 3) and ST648 (n = 1). The remaining isolates that did not co-carry *bla*_CTX-M-15_ belonged to ST410 (n = 2) and ST361 (n = 1).

The plasmid-encoded mobile colistin resistance *mcr-1.1* genes for colistin resistance were detected in only 2 (0.3%) of the *E. coli* isolates; 5 isolates of *K. pneumoniae* carried *mcr-8.1* in 3 isolates and *mcr-9* in 1 isolate distributed among ST15, ST14, ST29, and ST16. One *K. pneumoniae* isolate carried *mcr-9* and *bla*_VIM-4_. Several other resistance determinants were detected (Figures 2, 3), many of which were carried on plasmid replicons (i.e., IncFIB [77.7%], IncFIA_1 (59.5%], and IncFIB(K)_1 [59.9%] for *E. coli* and IncFIB(K)_1 [59.9%] and IncFII(pKP91)1 [56.6%] for *K. pneumoniae*). Of note, most *K. pneumoniae* isolates harboring carbapenemase-resistance genes had multiplasmid replicons ranging from 2 to 9 replicons per isolate, especially the self-transmissible IncFII-IncFIB plasmid carrying the *bla*_NDM-1_ gene. Variability in the surveillance strategies and clinical characteristics of patients between countries could have skewed the between-country isolate genomic characteristics and numbers of *E. coli* and *K. pneumoniae* isolates in the different populations.

## Discussion

The increasing spread of high-risk clones of *E. coli* and *K. pneumoniae* constitutes a serious threat for managing infections caused by those bacteria (*5*) to civilian and military populations, which often operate in harsh environments that increase their exposure to MDR pathogens. The population structure revealed high genetic diversity of STs and resistance determinants in the different countries. The *E. coli* population was dominated by ST131 in all 3 countries, consistent with its global dominance regardless of source (*13*), and was followed by ST1193, a high-risk clone that recently diverged from ST131.

Emerging *E. coli* ST1193 in Uganda and Kenya are frequently associated with extra-intestinal community-acquired urinary tract (*14*) and bloodstream infections, often with quinolone resistance-determining region mutations, ESBL *bla*_CTX-M_ genes, and IncF plasmids (*15*). Of note, potential zoonotic STs (ST10, ST95, and ST117) were detected, some of which are common in food animals (*16*–*19*) and known to carry an abundance of virulence factors and pathogenic potential that enable them to transmit, persist, and adapt to different hosts and environments (*17*).

*K. pneumoniae* isolates ST39 and ST17 were found mainly in East Africa countries and have previously been described in Kenya and Uganda (*20*,*21*). ST17 has been associated with regional outbreaks in Tanzania and Kenya and is prone to causing hospital outbreaks, making it an ST to monitor closely (*22*,*23*). In Jordan, high-risk CG14 (ST14) and CG147 (ST147) were dominant compared with East Africa countries, which could be associated with Jordan’s surveillance being focused on nosocomial infections (*24*), as well as the MDR CG258, which indicate the unique threats in Jordan. ST25, identified in MDR isolates from Kenya and Jordan, is concerning because of its reported hypermucoviscous phenotype and virulence-AMR convergence, resulting in poor clinical outcomes, although we did not detect that convergence in our study (*25*,*26*).

We identified a high diversity of resistance mechanisms; about half of the isolates carried an ESBL gene, mainly because of the extensively distributed *bla*_CTX-M-15_ gene, which was more prevalent among *K. pneumoniae* than among *E. coli.* Our study also detected several carbapenemase genes, primarily in *K. pneumoniae* isolates. Jordan reported more carbapenemase-resistance isolates than did the East Africa countries, similar to previous reports of high carbapenemase-resistance levels in Jordan (*24*) and India, which reported 30%–35% and co-expression of NDM and OXA-48 in 15.3% of carbapenemase-resistance isolates (*27*).

The increased resistance to last-line antimicrobial drugs (i.e., carbapenems and third- and fourth-generation cephalosporins) is concerning amid the increased excess, access, and misuse of antimicrobial drugs. The increase in mobile genetic elements that mobilize and spread resistance determinants further enhances spread. IncF and Col plasmids were the most common plasmid replicons among the MDR isolates; IncF plasmids are considered the more relevant contributors to the spread of AMR (*28*,*29*).

Overall, our study highlights the emergence and threat of genetically diverse high-risk MDR clones of 2 of the most critical groups of MDR bacteria causing severe infections with limited treatment options. The abundance of global high-risk STs bearing resistance genes indicates their effective dissemination, the potential for intraspecies and interspecies transmission of resistance genes, and emergence of new high-risk clones. To curtail the threat, continuous surveillance to monitor spread and emergence of dangerous clones is critical for supporting effective preventive measures and tailored therapies to match the regional and global risk to public and military health.

Appendix Additional information for study of genomic epidemiology of multidrug-resistant *Escherichia coli* and *Klebsiella pneumoniae*, Kenya, Uganda, and Jordan.

## References

[1] El Haddad L, Harb CP, Gebara MA, Stibich MA, Chemaly RF. A systematic and critical review of bacteriophage therapy against multidrug-resistant ESKAPE organisms in humans. Clin Infect Dis. 2019;69:167–78. 10.1093/cid/ciy947 30395179

[2] Jonas OB, Irwin A, Berthe FCJ, Le Gall FG, Marquez Patricio V. Drug-resistant infections: a threat to our economic future (vol. 2): final report. Washington (DC): World Bank Group; 2017.

[3] Poudel AN, Zhu S, Cooper N, Little P, Tarrant C, Hickman M, et al. The economic burden of antibiotic resistance: A systematic review and meta-analysis. PLoS One. 2023;18:e0285170. 10.1371/journal.pone.0285170 37155660 PMC10166566

[4] Baker S, Thomson N, Weill FX, Holt KE. Genomic insights into the emergence and spread of antimicrobial-resistant bacterial pathogens. Science. 2018;360:733–8. 10.1126/science.aar3777 29773743 PMC6510332

[5] Mathers AJ, Peirano G, Pitout JDD. The role of epidemic resistance plasmids and international high-risk clones in the spread of multidrug-resistant Enterobacteriaceae. Clin Microbiol Rev. 2015;28:565–91. 10.1128/CMR.00116-14 25926236 PMC4405625

[6] Magiorakos AP, Srinivasan A, Carey RB, Carmeli Y, Falagas ME, Giske CG, et al. Multidrug-resistant, extensively drug-resistant and pandrug-resistant bacteria: an international expert proposal for interim standard definitions for acquired resistance. Clin Microbiol Infect. 2012;18:268–81. 10.1111/j.1469-0691.2011.03570.x 21793988

[7] Centers for Disease Control and Prevention. CDC/NHSN surveillance definitions for specific types of infections [cited 2024 Apr 22]. https://www.cdc.gov/nhsn/pdfs/pscmanual/17pscnosinfdef_current.pdf

[8] Clinical and Laboratory Standards Institute. Performance standards for antimicrobial disk susceptibility tests, 14th ed. CSLI M02. Wayne (PA): The Institute: 2024.

[9] Mills EG, Martin MJ, Luo TL, Ong AC, Maybank R, Corey BW, et al. A one-year genomic investigation of *Escherichia col*i epidemiology and nosocomial spread at a large US healthcare network. Genome Med. 2022;14:147. 10.1186/s13073-022-01150-7 36585742 PMC9801656

[10] Feldgarden M, Brover V, Haft DH, Prasad AB, Slotta DJ, Tolstoy I, et al. Validating the AMRFinder tool and resistance gene database by using antimicrobial resistance genotype-phenotype correlations in a collection of isolates. Antimicrob Agents Chemother. 2019;63:e00483–19. 10.1128/AAC.00483-19 31427293 PMC6811410

[11] Hunt M, Mather AE, Sánchez-Busó L, Page AJ, Parkhill J, Keane JA, et al. ARIBA: rapid antimicrobial resistance genotyping directly from sequencing reads [cited 2024 Apr 24]. https://www.microbiologyresearch.org/content/journal/mgen/10.1099/mgen.0.000131 10.1099/mgen.0.000131 PMC5695208 29177089

[12] Ciccarelli FD, Doerks T, von Mering C, Creevey CJ, Snel B, Bork P. Toward automatic reconstruction of a highly resolved tree of life. Science. 2006;311:1283–7. 10.1126/science.1123061 16513982

[13] Ding Y, Zhang J, Yao K, Gao W, Wang Y. Molecular characteristics of the new emerging global clone ST1193 among clinical isolates of *Escherichia col*i from neonatal invasive infections in China. Eur J Clin Microbiol Infect Dis. 2021;40:833–40. 10.1007/s10096-020-04079-0 33118058

[14] Pitout JDD, Peirano G, Chen L, DeVinney R, Matsumura Y. *Escherichia coli* ST1193: Following in the Footsteps of *E. coli* ST131. Antimicrob Agents Chemother. 2022;66:e0051122. 10.1128/aac.00511-22 35658504 PMC9295538

[15] Wu J, Lan F, Lu Y, He Q, Li B. Molecular characteristics of ST1193 clone among phylogenetic group B2 non-ST131 fluoroquinolone-resistant *Escherichia coli.* Front Microbiol. 2017;8:2294. 10.3389/fmicb.2017.02294 29209300 PMC5702334

[16] Byarugaba DK, Wokorach G, Alafi S, Erima B, Najjuka F, Mworozi EA, et al. Whole genome sequencing reveals high genetic diversity, diverse repertoire of virulence-associated genes and limited antibiotic resistance genes among commensal *Escherichia coli* from food animals in Uganda. Microorganisms. 2023;11:1868. 10.3390/microorganisms11081868 37630428 PMC10457813

[17] Haley BJ, Salaheen S, Kim SW, Van Kessel JA. Virulome analysis of *Escherichia coli* ST117 from bovine sources identifies similarities and differences with strains isolated from other food animals. PLoS One. 2024;19:e0296514. 10.1371/journal.pone.0296514 38175844 PMC10766182

[18] Mora A, Viso S, López C, Alonso MP, García-Garrote F, Dabhi G, et al. Poultry as reservoir for extraintestinal pathogenic *Escherichia coli* O45:K1:H7-B2-ST95 in humans. Vet Microbiol. 2013;167:506–12. 10.1016/j.vetmic.2013.08.007 24008093

[19] Nandanwar N, Janssen T, Kühl M, Ahmed N, Ewers C, Wieler LH. Extraintestinal pathogenic *Escherichia coli* (ExPEC) of human and avian origin belonging to sequence type complex 95 (STC95) portray indistinguishable virulence features. Int J Med Microbiol. 2014;304:835–42. 10.1016/j.ijmm.2014.06.009 25037925

[20] Muraya A, Kyany’a C, Kiyaga S, Smith HJ, Kibet C, Martin MJ, et al. Antimicrobial resistance and virulence characteristics of *Klebsiella pneumoniae* Isolates in Kenya by whole-genome sequencing. Pathogens. 2022;11:545. 10.3390/pathogens11050545 35631066 PMC9144577

[21] Byarugaba DK, Erima B, Wokorach G, Alafi S, Kibuuka H, Mworozi E, et al. Genome analysis of *Klebsiella pneumoniae* reveals international high-risk pandemic MDR clones emerging in tertiary healthcare settings in Uganda. Pathogens. 2023;12:1334. 10.3390/pathogens12111334 38003798 PMC10674604

[22] Sonda T, Kumburu H, van Zwetselaar M, Alifrangis M, Mmbaga BT, Aarestrup FM, et al. Whole genome sequencing reveals high clonal diversity of *Escherichia coli* isolated from patients in a tertiary care hospital in Moshi, Tanzania. Antimicrob Resist Infect Control. 2018;7:72. 10.1186/s13756-018-0361-x 29977533 PMC5992844

[23] Henson SP, Boinett CJ, Ellington MJ, Kagia N, Mwarumba S, Nyongesa S, et al. Molecular epidemiology of *Klebsiella pneumoniae* invasive infections over a decade at Kilifi County Hospital in Kenya. Int J Med Microbiol. 2017;307:422–9. 10.1016/j.ijmm.2017.07.006 28789913 PMC5615107

[24] Hammour KA, Abu-Farha R, Itani R, Karout S, Allan A, Manaseer Q, et al. The prevalence of carbapenem resistance gram negative pathogens in a tertiary teaching hospital in Jordan. BMC Infect Dis. 2023;23:634. 10.1186/s12879-023-08610-4 37759305 PMC10523830

[25] Cejas D, Elena A, Guevara Nuñez D, Sevillano Platero P, De Paulis A, Magariños F, et al. Changing epidemiology of KPC-producing *Klebsiella pne*umoniae in Argentina: Emergence of hypermucoviscous ST25 and high-risk clone ST307. J Glob Antimicrob Resist. 2019;18:238–42. 10.1016/j.jgar.2019.06.005 31202977

[26] Pei N, Li Y, Liu C, Jian Z, Liang T, Zhong Y, et al. Large-scale genomic epidemiology of *Klebsiella pneumoniae* identified clone divergence with hypervirulent plus antimicrobial-resistant characteristics causing within-ward strain transmissions. Microbiol Spectr. 2022;10:e0269821. 10.1128/spectrum.02698-21 35416698 PMC9045374

[27] Jaggi N, Chatterjee N, Singh V, Giri SK, Dwivedi P, Panwar R, et al. Carbapenem resistance in *Escherichia coli* and *Klebsiella pneumoniae* among Indian and international patients in North India. Acta Microbiol Immunol Hung. 2019;66:367–76. 10.1556/030.66.2019.020 31438725

[28] Pitout JDD, Chen L. The significance of epidemic plasmids in the success of multidrug-resistant drug pandemic extraintestinal pathogenic *Escherichia coli.* Infect Dis Ther. 2023;12:1029–41. 10.1007/s40121-023-00791-4 36947392 PMC10147871

[29] Montgomerie JZ. Epidemiology of *Klebsiella* and hospital-associated infections. Rev Infect Dis. 1979;1:736–53. 10.1093/clinids/1.5.736 396632

