# Supplementary material for: Genomic Epidemiology of Multidrug-Resistant Escherichia coli and Klebsiella pneumoniae in Kenya, Uganda, and Jordan
Source: Emerg Infect Dis. 2024 Nov;30(Suppl 2):S33–40. doi: 10.3201/eid3014.240370 (PMC11559566; doi:10.3201/eid3014.240370)
Supplement: Appendix — Additional information for study of genomic epidemiology of multidrug-resistant Escherichia coli and Klebsiella pneumoniae, Kenya, Uganda, and Jordan. [file 24-0370-Techapp-s1.pdf]

Article DOI: https://doi.org/10.3201/eid3014.240370

EID cannot ensure accessibility for supplementary materials supplied by authors. Readers who have difficulty accessing supplementary content should contact the authors for assistance.

Genomic Epidemiology of Multidrug-Resistant *Escherichia coli* and *Klebsiella pneumoniae* in Kenya, Uganda, and Jordan

Appendix

Sampling strategy per country

In Jordan, the program was established in 2011 as a joint and active prospective surveillance strategy for Healthcare associated Infections and antimicrobial resistance conducted through a collaboration between the Ministry of Health (MOH) and the U.S. Naval Medical Research Unit EURAFCENT(NAMRU-EAC). To date, the program has been implemented across five governorates with the participation of nine hospitals: five public Ministry of Health hospitals, three hospitals affiliated with the private sector, and one university teaching hospital. Bacterial isolates were obtained from clinical samples of inpatients identified with four infections: surgical site infections (SSIs), bloodstream infections (BSIs), urinary tract infections (UTIs), and pneumonia. Infections were identified as Healthcare associated infections using CDC's National Healthcare Safety Network case definitions. Infections that were present on admission were classified as community-acquired infections.

In Kenya, a hospital-based antimicrobial resistant surveillance program has been conducted since 2015 in 12 hospitals across 7 counties (Kisumu, Malindi, Lamu, Kericho, Nairobi, Busia, Kisii). The hospitals are public sub-county and county hospitals (n = 8) and military hospitals (n = 4). The main focus of the study is to identify the major bacterial causes of infections and estimate the prevalence, AST profiles, and associated/risk factors for military relevant multidrug resistant organisms (MDROs) and World Health Organization (WHO) priority pathogens. The surveillance study targeted community- and hospital-acquired skin and soft tissue, wounds, surgical site, urine, throat, and blood infections from in-patient and out-

patient populations above 2 months of age. An active patient recruitment approach was adopted to capture robust epidemiologic data combined with a passive surveillance strategy where ESKAPE-E isolates are obtained directly from hospitals with active microbiological laboratories but with limited epidemiologic data captured. The infections are classified as health-care associated or community-acquired infections based on the CDC definition (*1*) where HAIs are those infections detected on day 3 or later of admission to the facility and CAIs are those infections detected before day 3 of admission.

In Uganda, the isolates were recovered under the antimicrobial surveillance program of Makerere University Walter Reed Project which has been running since 2012 to the present. Samples were collected from patients attending healthcare services from four hospitals (General Military Hospital Bombo, Bwera General Hospital, Gulu Regional Referral Hospital, and Kiruddu National Referral Hospital) between 2015 to 2022 from both outpatients and inpatients. Isolates were obtained from the hospital laboratories for more detailed phenotypic analysis at the MUWRP reference laboratory.

MDR Isolates from the three countries were identified per the GEIS submission guidelines using the recommendations and tables of Magiorakos et al. (2012) (*2*) and were sent for genomic sequencing to the Walter Reed Army Institute of Research for enrollemnt in The Multidrug-Resistant Organism Repository and Surveillance Network (MRSN).

References

1. Centers for Disease Control and Prevention. CDC/NHSN surveillance definitions for specific types of infections [cited 2024 Apr 22]. https://www.cdc.gov/nhsn/pdfs/pscmanual/17pscnosinfdef_current.pdf

2. Magiorakos AP, Srinivasan A, Carey RB, Carmeli Y, Falagas ME, Giske CG, et al. Multidrug-resistant, extensively drug-resistant and pandrug-resistant bacteria: an international expert proposal for interim standard definitions for acquired resistance. Clin Microbiol Infect. 2012;18:268–81. PubMed https://doi.org/10.1111/j.1469-0691.2011.03570.x

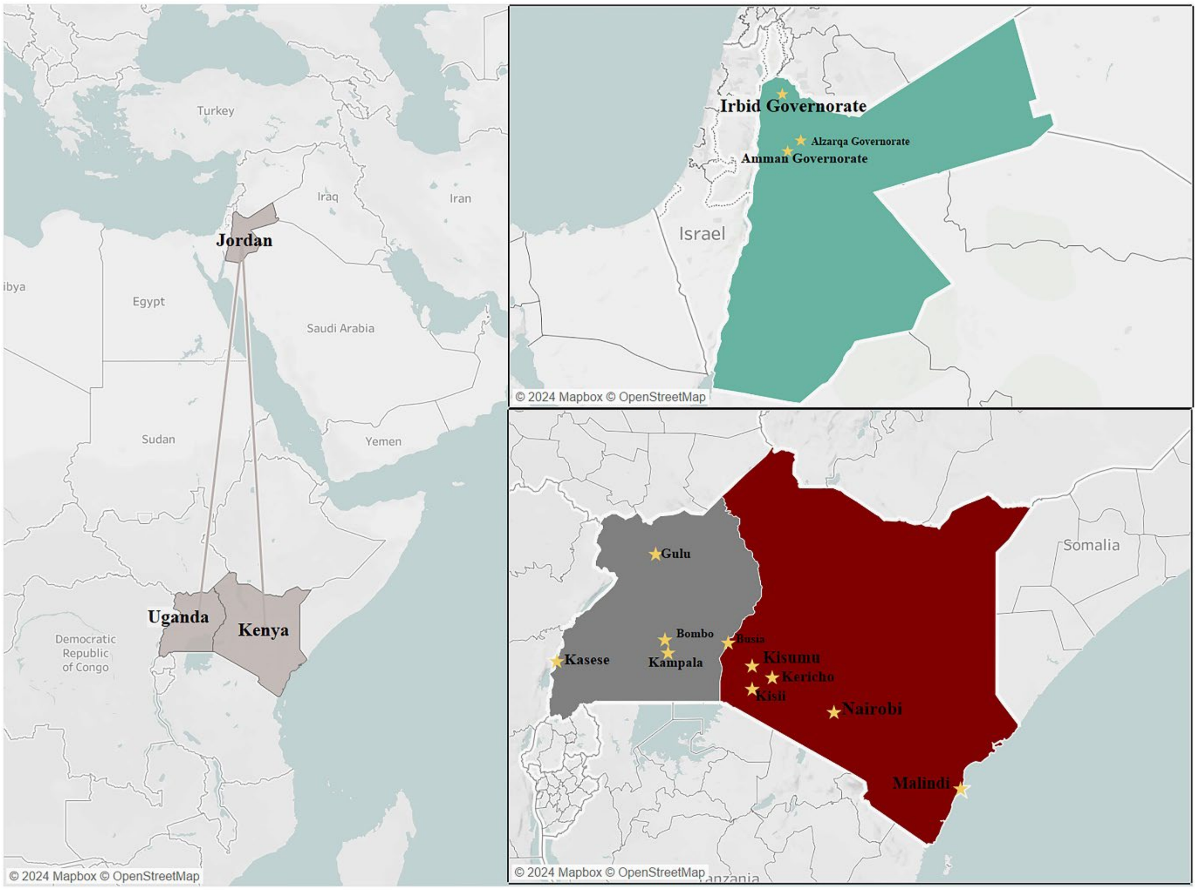

Appendix Figure 1. Map indicating relative geographic location of participating countries and hospitals: Varied geographic location of participating countries Jordan, Kenya, and Uganda (gray) and participating hospitals (stars) in Jordan and East Africa (Tableau Desktop Professional Edition 2022.4).

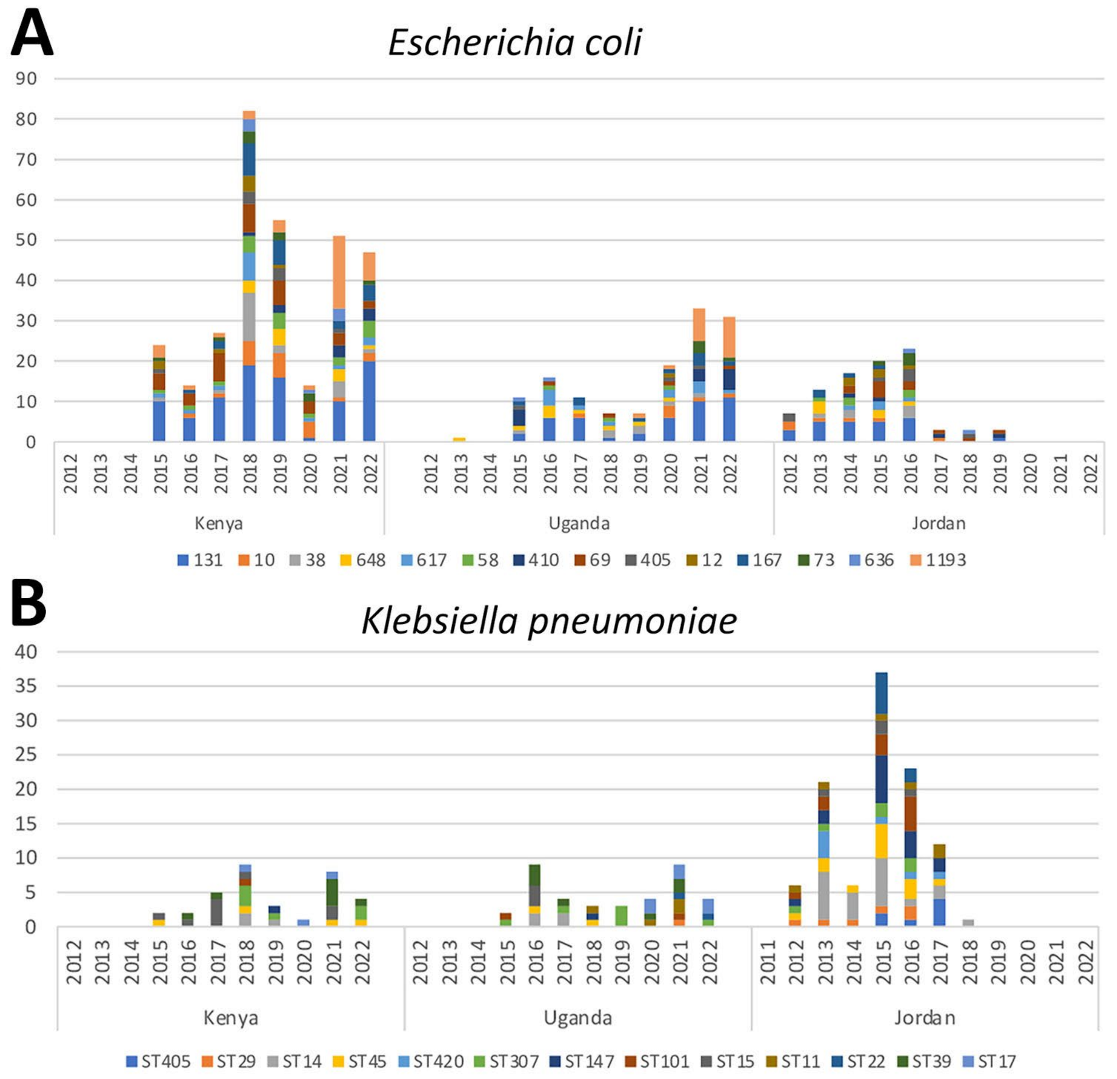

Appendix Figure 2. Annual proportions of high-risk *Escherichia coli* and *Klebsiella pneumoniae* sequence types across Jordan, Kenya, and Uganda over the 10-year period 2012–2022. ST, sequence type.
